# Assessing an eye injury patient

**Published:** 2015

**Authors:** Dorothy Mutie, Nyawira Mwangi

**Affiliations:** Consultant Ophthalmologist: Jaramogi Oginga Odinga Teaching and Referral Hospital, Kisumu, Kenya. Email: **d_mutie@yahoo.com**; Principal Lecturer: Ophthalmology Programmes, Kenya Medical Training College, Nairobi, Kenya. Email: **nyawiramwangi@yahoo.com**

**Figure:**
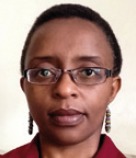
Dorothy Mutie

**Figure:**
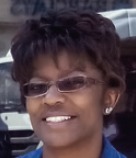
Nyawira Mwangi

Eye injuries are common, occurring either as isolated injuries or as part of head or facial injuries.

Health workers at primary level or in a trauma department should have a good knowledge of the presentation and management of ocular injuries. Health workers must be able to skillfully handle injuries to ocular structures in a way that aims to restore vision and prevent further loss of vision.

This article discusses the practical steps that should be taken at first contact with the patient and highlights the level of urgency of referral.

## First things first

Your first priority is to manage the anxiety and pain of the person with an eye injury (see article on page 50). Pain has been suggested as a factor in development of post-traumatic stress disorders (PTSD).^1^ For the patient with an eye injury:

- Reassure them of your intention to do your very best.
- Let children stay with their caregiver as much as possible.
- Even when the injury looks very bad, avoid giving that indication at the beginning, either by what you say to patients, what you say to other staff members, or through your facial expressions.
- Treat patients with courtesy, even if the cause of their injury is as the result of a fight.
- Give oral analgesics as needed.

## Dos

- Use a moist sterile dressing to cover the eye (with a shield for additional protection).
- Prevent secondary injuries, particularly infections, through aseptic techniques and appropriate use of antibiotics.
- Assess both eyes, even if the injury is unilateral.
- Document all the findings and procedures.
- Monitor visual outcome.
- Plan for rehabilitation of the patient.

## Don'ts

- Do not assume that a visual acuity of 6/6 excludes serious eye complications.
- Do not delay irrigation in chemical injuries.
- Do not delay referral.
- Do not manage eye injuries in a patient who is not stabilised: life-threatening conditions must be addressed first.
- Do not touch or otherwise manipulate an eye with rupture or perforating injury.
- Do not prescribe topical anaesthetic.
- Do not pull out a protruding foreign body.
- Do not use traditional eye medicines.

Mechanical injuries involving the eyeball can be classified using the Birmingham Eye Trauma Terminology System (BETTS), which applies only to mechanical eye injuries. Chemical and thermal injuries are dealt with separately. BETTS is a practical guide to classifying eye injuries. It ensures that all the health workers involved in the care of the same patient have a consistent understanding of the type of injury. Further, it helps to ensure that there is uniformity, which enables comparison of data in future audits and research (see article on page 43).

## What you need

**Essential (the basics)**

- Visual acuity chart
- Torch
- Cotton buds
- Lid speculum
- Eye shield (plastic/metallic)
- Local anaesthetic drops
- Antiseptic, e.g. iodine solution
- Tetanus toxoid
- Analgesics
- Irrigating fluid
- Topical anaesthetic drops
- Gloves for examination

**Additional (in an ideal scenario)**

- Slit lamp
- Tonometer
- Magnifying loupe
- Direct ophthalmoscope
- Eye pads
- Fluorescein strips
- Litmus paper
- Cyclopegic drops
- Topical and systemic antibiotics

## Initial assessment

The ABCDE approach to the evaluation and treatment of patients with potentially life-threatening injuries should be followed:

- **A**irway with cervical spine protection
- **B**reathing and ventilation
- **C**irculation
- **D**isability (using Glasgow Coma Scale and pupillary assessment)
- **E**xposure and Environment control.

Patients with serious non-ocular injuries or unstable vital signs should be managed in a trauma facility. Following stabilisation the specific assessment of ocular injuries can proceed.

## History

In the following eye injuries, the health worker needs to get a quick description of what happened, institute immediate measures and obtain a detailed history later.

1. Suspected chemical injury: immediate irrigation as described below.
2. Active bleeding: arrest bleeding and pad the eye.
3. Severe pain, especially in children: give analgesia to enhance patient comfort.

For all other eye injury patients, a detailed history should betaken, including:

- **Age and occupation**
- **Presenting symptoms.** Which eye is affected? Is it both eyes? Is there diplopia (double vision)?
- **Source and mechanism of injury.** Chemical and thermal injuries need to be identified and treated immediately. Blunt objects (such as closed fists or blocks of wood) are more likely to cause rupture of the eyeball, whereas sharp objects (such as knives) are likely to cause lacerations. Inert intraocular foreign bodies (such as glass, stone or plastic) cause less reaction than metals (copper, aluminium, lead, iron). Plant material (wood or vegetable matter) are poorly tolerated. If an intra-ocular foreign body (IOFB) is suspected, obtain information on its composition: organic (plant matter) or inorganic (metal), magnetic or not, and any possible chemical property, such as acidic or alkaline.
- **Time of the injury.** This helps to determine the treatment strategy, as a quiet eye with a sealed 1-week-old corneal laceration may be managed conservatively, whereas an acute injury requires surgical intervention.
- **Place of injury.** Where did the injury occur?
- **Events surrounding the injury.** Was the injury accidental or non-accidental and was protective gear (e.g. protective eyewear or seat belts) in use, where appropriate?
- **Any steps taken to manage the injury prior to presentation at health facility.** These include irrigation, use of any medication, and removal of foreign body at home.
- **Previous ocular history.** This includes the vision before the injury, use of contact lenses, previous trauma or surgery, and current medication.
- **Current and previous medical history.** Existing conditions such as diabetes mellitus, hypertension, bleeding disorders and allergies need to be identified.

## The examination

**NOTE:** If you suspect an open globe injury, stop. You can make it worse by examining it, causing increased prolapse of ocular contents. Refer the patient to theatre for examination under anaesthesia.

The patient may be examined in an upright position if it is possible to do so. If it is difficult to open the eyes, lying down may make it easier. Children should generally sit on their parents' laps or lie down if retraction of the lids may be required.

**Figure 1.**
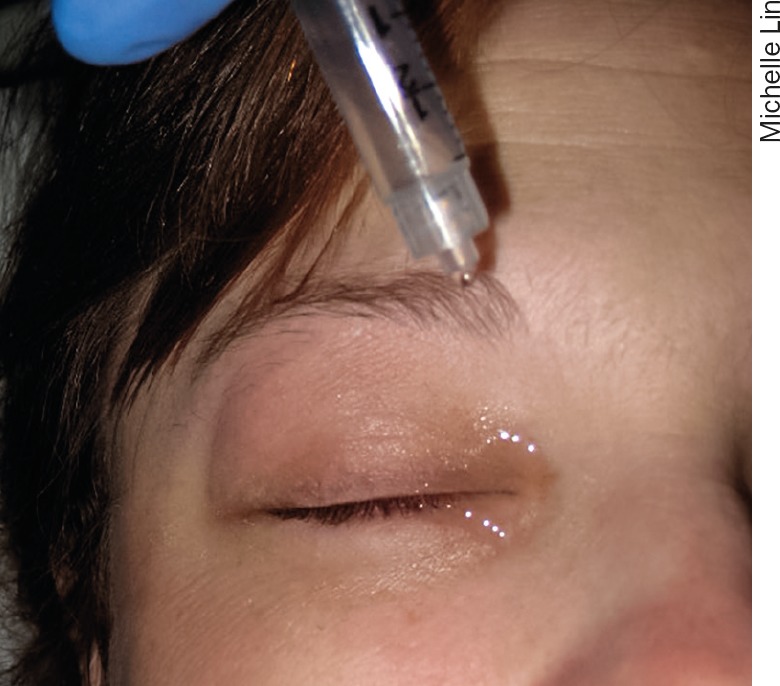
Instilling topical anaesthetic when the eyelids cannot be opened

If the patient has difficulty opening the eyes, topical anaesthetic drops helps to reduce the pain and allows for examination. Ask the patient to tilt their head backwards or to lie down. Instil a few drops on the medial canthus area of the affected eye (i.e. nearest the nose) and ask the patient to blink briefly. This will allow some of the anaesthetic to seep into the eye and provide relief (Figure 1). Retracting eyelids aids in observation of the rest of the structures and in irrigation. Use lid retractors or a lid speculum. If these are not available, use bent paper clips.

### Step 1: Visual examination

**Figure 2:**
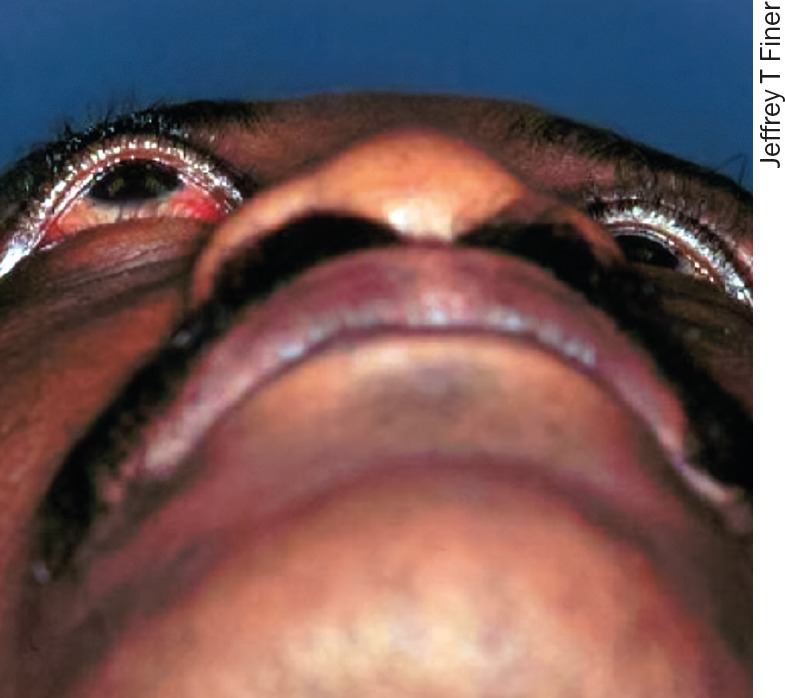
Proptosis (right eye).

**Figure 3.**
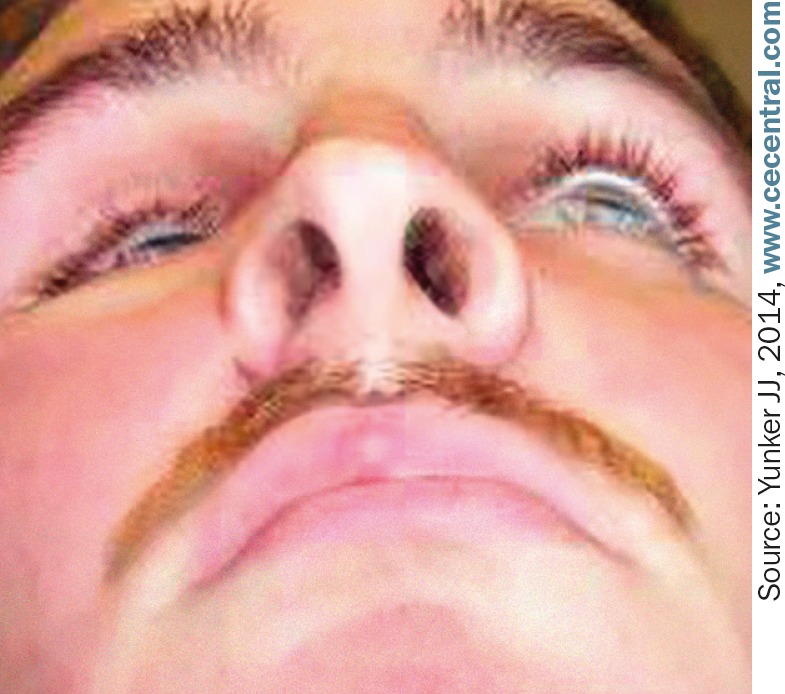
Traumatic enophthalmos (right eye).

- **Inspection.** Record the location, size and appearance of obvious injuries such as lacerations, swelling (contusions) or foreign bodies (FB). Note any proptosis (Figure 2) or enophthalmos (Figure 3).
- **Visual acuity.** Record the visual acuity for each eye (presenting vision, and vision with pinhole).
- **Orbital wall.** Should be palpated for crepitus or bony deformity.
- **Ocular motility.** Should be assessed in the cardinal directions of gaze (vertical up-down, horizontal right to left, diagonal left to right and right to left)
- **Visual fields.** May be assessed by confrontation method.
- **Adnexae.** Lid defects (ptosis, lacerations) should be noted. Canaliculi injury should be suspected in medial eyelid injuries.
- **Eyeball.** If the lids of both eyes can open, assess whether both eyeballs are of the same size. A smaller eye could mean a blow-out fracture; or the larger eye could mean bleeding behind the eyeball or orbit.

**Figure 4:**
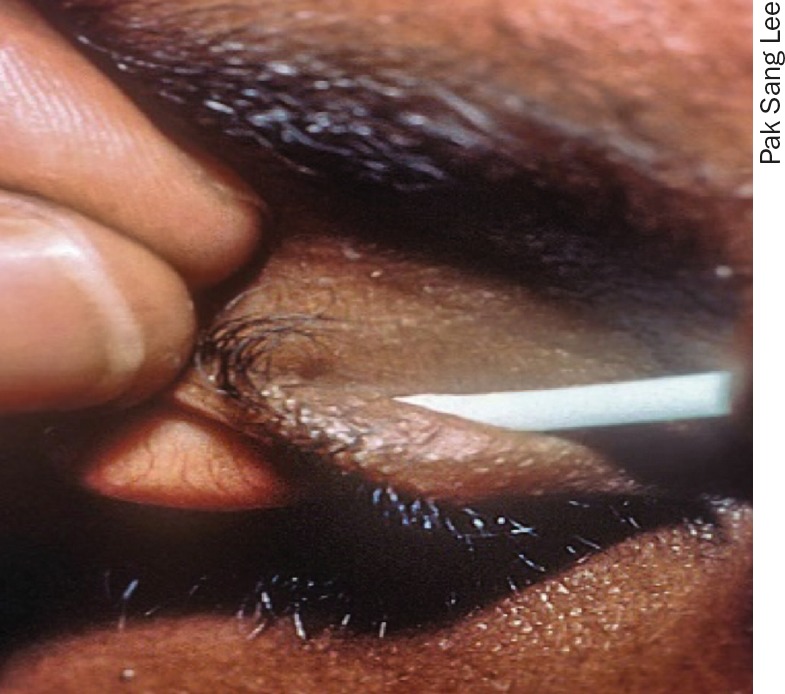
Everting the upper eyelid using a cotton bud.

**Figure 5:**
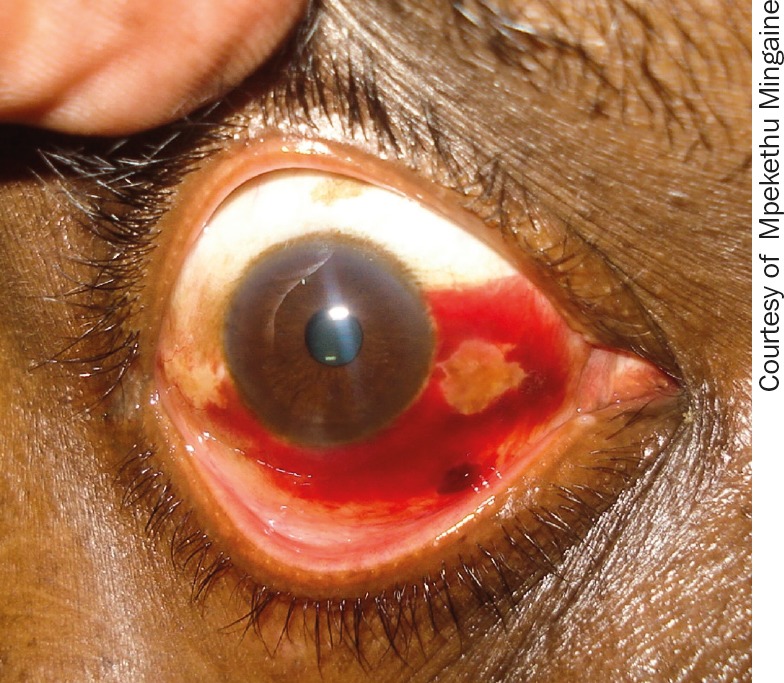
Sub-conjunctival haemorrhage.

**Figure 6.**
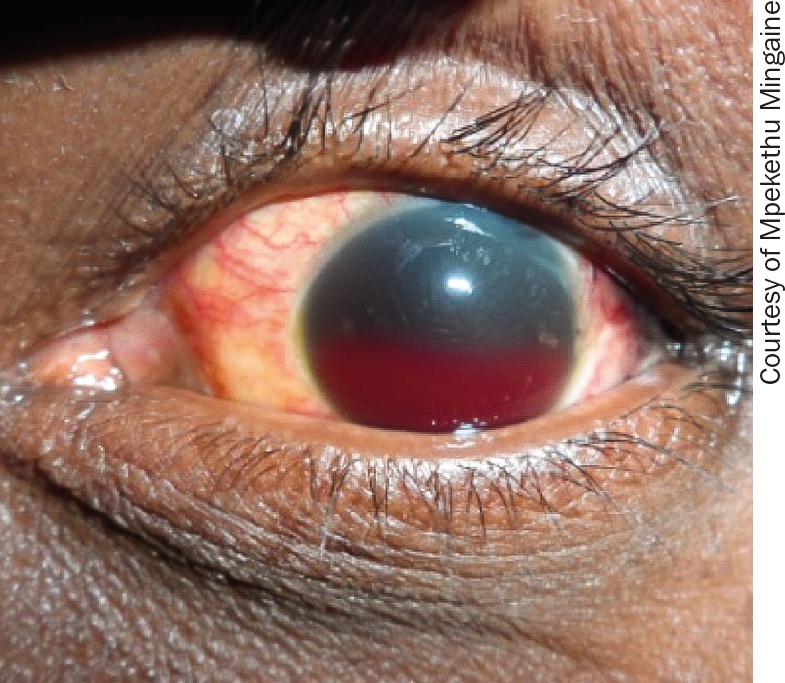
Hyphaema.

### Step 2: Using a source of light

For the next steps, use a source of light. Where there is a slit lamp, use it, provided that the patient is comfortable.

- **Examining the upper and lower fornix.** Ask the patient to look up as you pull the lower eyelid to expose the lower fornix. The upper fornix and tarsal conjunctiva are observed better if the lid is double everted using a Desmarres lid retractor. A cotton bud may be used to evert the lid (Figure 4) but the upper fornix will be difficult to visualise. Look out for foreign bodies and lacerations.
- **Conjunctiva.** Note any sub-conjunctival haemorrhage (Figure 5) and its furthest extent. This is important for follow up as well as to rule out hidden scleral lacerations.
- **Cornea and sclera.** These may have full length or lamellar lacerations, with or without uveal prolapse (the iris popping out through the cornea). Corneal staining with fluorescein can reveal scratch marks from a hidden conjunctival FB.
- **Anterior chamber.** If there is blood in the anterior chamber (hyphaema), estimate the level of the hyphaema while the patient is in an upright position (Figure 6). If lens matter (whitish material in the anterior chamber) is noted, **refer very urgently (within 1–2 hours).**
- **Pupils.** Note the size and shape, and the direct and consensual light reaction, as well as the relative afferent pupillary defect (RAPD).
- **Lens.** In some instances, it may be possible to observe a cataractous lens without lens rupture.
- **Intraocular pressure (IOP).** This should also be assessed where there is closed globe injury (no full thickness wound of the eyeball). Low pressure may indicate occult globe rupture or retinal detachment. High pressure can be expected immediately after contusion to the globe.
- **Posterior segment.** Pupils may be dilated for fundoscopy if intraocular pressure (IOP) is normal and there is no expanding hyphaema.
