# Supplementary material for: Assessing an eye injury patient
Source: Community Eye Health. 2015;28(91):46–8. (PMC4790160)
Supplement: Related texts

# Brief report: A note on referral

**Published:** 2015

The existing referral system should be used, ensuring that patients are referred to a secondary or tertiary level facility that has doctors with the skills (and equipment) to handle eye injuries appropriately.

Talk to patients about their injury and the reason for the referral (see article on page 50). Write down the most important information if possible. Check that the patient and her or his accompanying person know where to go, and how urgently. Clean as much blood and debris as is possible from around the injured area, even if referring immediately.

In this article and the one following:

- Refer **immediately** means the patient should be seen by an appropriately qualified person within minutes.
- Refer **very urgently** means she or he should be seen within 1–2 hours
- Refer **urgently** means the patient should be seen within 24 hours.
